# Network models provide insight into how oriens-lacunosum-moleculare (OLM) and bistratified cell (BSC) interactions influence local CA1 theta rhythms

**DOI:** 10.1186/1471-2202-15-S1-P42

**Published:** 2014-07-21

**Authors:** Katie A Ferguson, Carey YL Huh, Bénédicte Amilhon, Sylvain Williams, Frances K Skinner

**Affiliations:** 1 Toronto Western Research Institute, University Health Network, Toronto, Ontario, M5T 2S8, Canada; 2 Physiology, University of Toronto, Toronto, Ontario, M5S 1A1, Canada; 3 Psychiatry, Douglas Mental Health University Institute, McGill University, Montreal, Quebec, H4G 1X6, Canada; 4 Medicine (Neurology), University of Toronto, Toronto, Ontario, M5S 1A1, Canada

## 

Although hippocampal theta, a 4-12 Hz rhythm associated with episodic memory, has been studied extensively, the cellular mechanisms underlying its generation are unclear. OLM cells have been considered pacemakers of local CA1 theta [1], but recent experimental work has disputed this role [2]. The complex interactions that OLM cells have with other cell types, such as bistratified cells (BSCs) [3], make their contribution to network rhythms difficult to determine experimentally. One can address this issue using mathematical network models, which allow one to explore the contribution of specific cell populations and network connectivity in a simplified setting, and make predictions to guide further experimental work. Thus, we created a network model that is tied to experimental work on both the cellular and network level, and explored how cell interactions affect the power of local oscillations.

We derived cellular properties from patch clamp recordings of fast-spiking parvalbumin-positive (PV+) interneurons – likely comprising basket cells (BCs), axo-axonic cells (AACs), and BSCs – and of somatostatin-positive putative OLM cells in the CA1 region of an intact hippocampus *in vitro*, and used these properties to constrain Izhikevich-type models of BCs/AACs, BSCs, and OLM interneurons. We constructed our network model with these individual cell models, and constrained network size, connectivity, and synaptic properties with experimental data. Experimental excitatory postsynaptic currents (EPSCs) recorded during endogenous CA1 theta oscillations were used to drive the various cell model populations, and we used a simple local field potential (LFP) model to integrate the effects of cell firing. To determine how the interactions between OLM cells and BSCs affect local theta rhythms, we explored how specific features of the network affected model LFP power. In addition, we simulated optogenetic experiments by silencing the OLM cell model population during the network rhythm.

Spike characteristics and firing behaviors in our network models approximated those determined experimentally. Our models distinguish between regimes in which OLM cells minimally or strongly affect the power of network oscillations, and predict that the dis-inhibitory effect of OLM cells on BSC to pyramidal cell interactions plays a critical role in the power of network theta oscillations. When OLM to BSC model connections are not too strong, the OLM cells’ direct influence on pyramidal cells balances with its indirect dis-inhibitory effect (through the BSCs). In this case, when the OLM cell population is silenced, there is a compensatory effect on network power, and thus minimal change in power. However, when these OLM to BSC connections are stronger, the dis-inhibition of pyramidal cells does not balance with their direct influence, and thus silencing OLM cells has a stronger effect. This does not change when we consider various distributions of strengths in which the cell populations affect the LFP. Thus, our network models are able to make particular predictions that can be tested with optogenetics.
